# Cognitive impairment in health care workers recovering from COVID-19 infection: a cross-sectional comparative study

**DOI:** 10.1186/s43045-022-00245-6

**Published:** 2022-10-17

**Authors:** Ahmed Khaled Abd-Elrazzak Omar, Salwa M. A. Dahesh, Doha El-Sayed Ellakwa, Mohamed Kamal Gomaa, Basma Abdulsamad, Rana Hanafy, Hanan G. Al Metwally, Ruqia Nour Edin Mohammad Mohammad, Samar Saleh Badawy, Rabab M. El Saleh, Mohammed E. Abdelhafiz, Abdalla Mohamed Gouda, Showikar Adel Saleh Seada, Marwa M. Amr, Yomna Asar, Roa Gamal Alamrawy

**Affiliations:** 1 Infectious Disease and Gastroenterology, Ministry of Health and Population, Cairo, Egypt; 2 Research Institute of Medical Entomology, General Organization for Teaching Hospitals and Institutes, Ministry of Health and Population, Cairo, Egypt; 3 Biochemistry and Molecular Biology Department, Faculty of Pharmacy, Al-Azhar University, Cairo, Egypt; 4 Rheumatology Department, Alazhar University, Cairo, Egypt; 5 Ministry of Health and Population, Cairo, Egypt; 6 Oncology Center, Mansoura University, Mansoura, Egypt; 7 Mamoura Psychiatric Hospital, General Secretariat of Mental Health and Addiction Treatment, Ministry of Health and Population, Alexandria, Egypt; 8 Neuroscience Research Master Candidate, Erasmus University Rotterdam, Rotterdam, the Netherlands

**Keywords:** COVID-19, Long-term symptoms, Persistent symptoms, Cognitive impairment, Cognitive function, Neuropsychology

## Abstract

**Background:**

The COVID-19 outbreak has infected people all over the world where many clinics are being constructed to diagnose and treat lingering symptoms or long COVID. Neurological and long-term cognitive consequences are very worrisome. Many of COVID-19’s neurological symptoms are likely the result of the body’s extensive immunological response to infection rather than the virus attacking the brain or nervous system directly. At the same time, the extent and type of COVID-19’s cognitive consequences are unknown. The goal of this study was to assess the cognitive functions of healthcare workers 2 weeks to 3 months after COVID-19 infection. Ninety-two healthcare workers participated in the study; 32 were post-COVID-19 cases, and 60 were healthy people (the comparison group). The cognitive functions of the participants were assessed using the Addenbrooke’s Cognitive Examination (ACE-III) test, which evaluated attention, memory, language, and visuospatial skills, as well as the Arabic version of the Patient Health Questionnaire Anxiety GAD-7 and Depression Assessments PHQ-9.

**Results:**

The study revealed that there was a highly significant direct correlation between post-infection with COVID-19 and scores of both anxiety and depression and an inverse correlation in the case of attention and memory. On the other hand, there is no statistical effect of post-COVID-19 on verbal fluency, language scores, and visio-spatial abilities. Using multiple linear regression, there was a powerful significant decrease effect of post-COVID-19 on memory scores controlling both anxiety and depression degrees (Beta = − 0.745, *P* < 0.001). Also, there was a strong negative correlation post-COVID-19 on attention scores controlling both anxiety and depression degrees (Beta = − 0.745, *P* < 0.001).

**Conclusions:**

The study showed a strong negative effect of post-COVID-19 on the attention and memory of patients. Furthermore, both anxiety and depression scores increased significantly among the post-COVID-19 patients.

## Background

The World Health Organization (WHO) declared coronavirus disease 2019 (COVID-19) an international public health emergency on January 30, 2020 [1]. SARS-CoV-2 coronavirus, a positive-sense RNA virus belonging to the Coronaviridae family, causes COVID-19, a highly contagious respiratory infection (Malik et al. 2020). As of July 2022, WHO reported over 535 million confirmed cases of COVID-19 including more than 6 million deaths [1].

Although the respiratory tract represents the primary affected organ in COVID-19 patients, other organs and systems had been involved and reported including cardiovascular, renal, and neurological systems [2]. Neuropsychiatric complications of COVID-19 have been reported progressively by multiple case reports and case series. Delirium, confusion, and neurocognitive abnormalities were some of the most common documented neurological problems in COVID-19 patients [3]. Depression, anxiety, post-traumatic stress disorder (PTSD), and insomnia were the common psychiatric disorders among COVID-19 patients [4–7]. By dissecting the molecular pathogenesis of SARS-CoV-2, different neuro-invasive mechanisms have been proposed [8]. Coronavirus infection of the central nervous system (CNS) could occur via olfactory mucosa, peripheral leukocytes adhering to endothelial cells of the blood-cerebrospinal fluid (CSF) barrier, or oxidative stress generated by brain-lung-heart interaction. These associated mechanisms result in the overproduction of pro-inflammatory cytokines leading to final demyelination and inflammation of neurons [4].

Since healthcare workers (HCWs) represent the front line of defense in this crisis, neuropsychiatric complications of COVID-19 among them represent a highly significant research topic. An increased risk of infection is expected among HCWs due to their close contact with infected patients, especially those with undiagnosed or subclinical cases [9]. According to a recently published meta-analysis, HCWs were reported to be exposed to a variety of long-term psychological stressors, such as anxiety (37%), sadness (36%), and insomnia (32%) [10]. However, studies are lacking in handling neuro-psychological complications other than depression, anxiety, and insomnia. Additionally, all quantitative studies were cross-sectional studies with short follow-up periods, which did not reflect COVID-19’s long-term mental health outcomes on HCWs. Also, the impact and severity of this epidemic vary greatly from one country to another [10].

The present study aimed to evaluate cognitive dysfunctions in Egyptian HCWs post-COVID-19 infection as attention, memory, language, and visuospatial skills in addition to anxiety and depression. Diagnosis of long-term COVID-19 cognitive dysfunctions will enable the implementation of diagnostic and therapeutic protocols necessary for the treatment and rehabilitation of affected HCWs. In turn, it will contribute in the reduction of their medical errors that affect the lives of their patients besides maintaining the health of HCWs, the first line of defense, against this pandemic.

## Methods

### Study design and study population

The study design was an observational cross-sectional comparative study, with a convenience sampling method used. Demographic and clinical features in a cohort of HCWs recovered from COVID-19 were investigated and compared with a comparative group of HCWs during the outbreak period (from June to October 2021). *Inclusion criteria* were the medical staff (physicians, dentists, pharmacists, nurses, and medical technicians) aged more than 18 years old and able to complete the test content independently. The study included the post-COVID-19 cases that have recovered from 2 weeks to 3 months during the study. The included patients have previously been diagnosed as COVID-19 cases and confirmed by polymerase chain reaction (PCR-for-COVID), a computed tomography scan (CT-chest), and routine tests battery besides symptoms like fever, cough, GIT symptoms, loss of smell or taste, and fatigue according to the COVID-19 protocol of the Ministry of Health and Population, Egypt. *The participated* HCWs worked at Hospitals of the Egyptian Ministry of Health and Population (Dekernes Hospital, Ras Sedr Hospital, El Hamool Hospital, Seidy Salem Hospital, Mansoura Oncology Center). The *exclusion criteria* were the HCWs with a history of mental disorders and current treatment for mental illnesses, such as taking antipsychotics, antidepressants, mood stabilizers, anti-epileptics, benzodiazepines, and other drugs that may interfere with the assessment. Also, HCWs had severe physical illnesses, serious suicidal thoughts, or hearing or visual impairments. Pregnant and lactating women were also excluded.

*Sample size calculations* were performed using SPSS software version 26 [11]. After careful examination of the literature, no references clarified the difference between the variables used in the present study. Based on this, the sample size was calculated on the ground during the study using the estimated means and standard deviation (SD) of the two groups. Epi Info software [12] was used for independent continuous groups assuming the mean ± SD of anxiety was equal to 9.8 ± 4.2 for the post-COVID-19 group and the mean equal to 6.4 for the comparative group at 0.05 alpha error and 95% power. When enrolment equals 2, the estimated sample size was determined as 30 and 60 for the case and comparative group, respectively.

### Measurement

Clinical interviews were carried out for collecting socio-demographic data and performing cognitive assessment by the Addenbrooke’s Cognitive Examination III (ACE-III) [13], anxiety assessment by the Arabic version of the Generalized Anxiety Disorder Scale (GAD-7) [14], and depression assessment by the Arabic version of the Patient Health Questionnaire (PHQ-9) [14]. Socio-demographic data were self-reported by the participants, including sex, age, residence, marital status, and occupation (physicians, dentist, pharmacist, chemist, nurse, or technician).

ACE-III is a brief cognitive battery that assesses various aspects of cognition and is one of the most widely used assessment tools in routine clinical practice. ACE-III is a 19-item tool that tests the five subdomains of cognition: attention and orientation, memory, verbal fluency, language, and visuospatial ability. ACE-III was easily applied by the trained researchers who interviewed the agreed participants face-to-face. The test was administered in 15–20 min, then scored and interpreted by an unbiased non-specialist. The total score of the examination is 100 and the higher scores indicate better cognitive functioning [13]. The Arabic version of the ACE-III showed high sensitivity and specificity [15, 16]. For assessing anxiety symptoms, the Arabic version of the Generalized Anxiety Disorder Scale (GAD-7) was used. A total score equal or more than 5 was considered having anxiety [17]. The validity and reliability of the Arabic version of GAD-7 have been confirmed where Cronbach’s alpha value for the internal insistency reliability was 0.763. Also, the validity and reliability of the Arabic versions of PHQ-9 were demonstrated where Cronbach’s alpha value for the internal consistency reliability was 0.857 [14, 17].

### Statistical analysis

Data of the two groups were collected and tabulated for statistical analysis by PC using the Epi Info and SPSS version 26 for windows software packages [11]. Both the homogeneity test and the Leven test were used. Descriptive analysis as the arithmetic mean and standard deviation of the age and scores of examinations of the participated HCWs were calculated. Student’s *t* test was used for comparing the continuous parametric data of the two groups. *Z*-test and chi-square were used for in-between proportions. Correlations and multiple linear regressions were used for estimating the relation between post-COVID-19 and various cognitive scores (Rosner, 2015). All statistical tests were interpreted in a two-tailed fashion at a cut-off value equal to 0.05.

### Ethics approval and consent to participate

The protocol of the study followed the professional ethics of the Declaration of Helsinki and was approved by Egypt’s ethical committee of the Minister of Health and Population (Com. No./Dec. No. 10-2021/12). All participants agreed and signed written informed consent forms before the interviews.

## Results

The total number of participants in this study was 92 healthcare workers; 16 (17.4%) were males and 76 (82.6%) were females. Their ages ranged from 22 to 54 years. The number of post-COVID-19 cases was 32 while the comparative group was 60 healthcare workers with different job categories: 17 physicians, 3 dentists, 36 pharmacists/chemists, 16 nurses, and 20 assistants. The results showed that there were no statistically significant differences between post-COVID-19 cases and the comparative group according to gender, job category (Table 1), mean age, verbal fluency, language scores, and visio-spatial abilities (Table 2). Although the mean score of Addenbrooke’s Cognitive Examination among post-COVID-19 cases was lower than that of the comparative group, the difference was still insignificant (*P* = 0.069). The mean scores of memories, attention, and were lower among post-COVID-19 cases than those among the comparative group where the differences were highly statistically significant (*p* < 0.01). On the other hand, the mean scores of the post-COVID-19 cases were significantly higher in the case of anxiety and depression (*p* < 0.05).

**Table 1:** Descriptive analysis of the post-COVID-19 cases and control group according to their gender and various job categories

Variables			COVID-19		***P*** value
			Control	Post-COVID-19 cases	
**Job category**	**Physician**	No.	8	9	**Exact Pearson chi-square** 0.266
		%	13.3%	28.1%	
	**Dentist**	No.	2	1	
		%	3.3%	3.1%	
	**Pharmacist/chemist**	No.	28	8	
		%	46.7%	25.0%	
	**Nurse**	No.	10	6	
		%	16.7%	18.8%	
	**Assistant**	No.	12	8	
		%	20.0%	25.0%	
	**Total**	No.	60	32	
		%	100.0%	100.0%	
**Gender**	**Male**	No.	12	4	0.407
		%	20.0%	12.5%	
	**Female**	No.	48	28	
		%	80.0%	87.5%	
	**Total**	No.	60	32	
		%	100.0%	100.0%

**Table 2:** A comparison between post-COVID-19 cases and a comparative group of healthcare workers according to their age and scores of attention, memory, verbal fluency, language, visio-spatial abilities, Addenbrooke’s cognitive examination, anxiety, and depression

Variables	Comparative group		Post-COVID-19 cases		*t*	*P* value
	Mean ± SD (*n* = 60)	SE	Mean ± SD (*n* = 32)	SE		
**Age (years)**	30.77 ± 6.021	0.777	32.88 ± 6.955	1.229	1.515	0.133
**Attention score**	17.63 ± 0.712	0.092	16.72 ± 1.708	0.302	3.612	*P* < 0.001**
**Memory score**	23.17 ± 2.101	0.271	16.72 ± 1.708	0..685	3.686	*P* < 0.001**
**Verbal fluency score**	11.43 ± 2.942	0.380	12.38 ± 2.673	0.473	1.508	0.135
**Language score**	24.87 ± 1.891	0.244	24.53 ± 1.606	0.284	0.852	0.396
**Visio-spatial abilities**	14.70 ± 1.522	0.196	14.97 ± 1.257	0.222	0.855	0.395
**Addenbrooke’s Cognitive Examination**	91.800 ± 5.70103	0.736	89.468 ± 5.967	1.054	1.838	0.069
**Anxiety score**	6.4667 ± 4.589	0.592	9.843 ± 4.258	0.752	3.445	0.001**
**Depression score**	8.300 ± 4.900	0.632	10.500 ± 5.224	0.923	1.965	0.048*

*P value < 0.05 (significant)

**P value < 0.01 (highly significant)

Concerning the degree of anxiety and depression, the lowest degrees were recorded among the comparative group while the post-COVID-19 cases group recorded the highest ones. The difference between the two groups was highly statistically significant (*p* < 0.01) (Table 3).

**Table 3:** A comparison between post-COVID-19 cases and the comparative group according to anxiety degree

Anxiety degree			COVID-19		Total
			The comparative group	Post-COVID-19 Case	
**Anxiety degree**	**No anxiety**	**No.**	**26**_**a**_	**3**_**b**_	**29**
		**%**	**43.3%**	**9.4%**	**31.5%**
	**Mild anxiety**	**No.**	**20**_**a**_	**11**_**a**_	**31**
		**%**	**33.3%**	**34.4%**	**33.7%**
	**Moderate anxiety**	**No.**	**10**_**a**_	**11**_**a**_	**21**
		**%**	**16.7%**	**34.4%**	**22.8%**
	**Severe anxiety**	**No.**	**4**_**a**_	**7**_**b**_	**11**
		**%**	**6.7%**	**21.9%**	**12.0%**
**Total**		**No.**	**60**	**32**	**92**
		**%**	**100.0%**	**100.0%**	**100.0%**

Exact Pearson chi-square = 14.546, *P* = 0.002

The letter *a* denotes a significant difference from the *b* column

The results revealed that there was a highly significant direct correlation between post-infection with COVID-19 and both scores and degrees of anxiety and depression and inverse correlation in the case of attention and memory (*p* < 0.001) (Tables 4, 5, 6, and 7).

**Table 4:** A comparison between post-COVID-19 cases and the comparative group according to depression degree

Depression degree			COVID-19		Total
			The comparative group	Post-COVID-19 cases	
**Depression degree**	**No depression**	**No.**	**18**_**a**_	**5**_**a**_	**23**
		**%**	**30.0%**	**15.6%**	**25.0%**
	**Mild depression**	**No.**	**22**_**a**_	**7**_**a**_	**29**
		**%**	**36.7%**	**21.9%**	**31.5%**
	**Moderate depression**	**No.**	**12**_**a**_	**11**_**a**_	**23**
		**%**	**20.0%**	**34.4%**	**25.0%**
	**Moderate to severe depression**	**No.**	**8**_**a**_	**5**_**a**_	**13**
		**%**	**13.3%**	**15.6%**	**14.1%**
	**Severe depression**	**No.**	**0**_**a**_	**4**_**b**_	**4**
		**%**	**0.0%**	**12.5%**	**4.3%**
**Total**		**No.**	**60**	**32**	**92**
		**%**	**100.0%**	**100.0%**	**100.0%**

Exact Pearson chi-square **=** 12.476, *P* = 0.012

The letter *a* denotes a significant difference from the *b* column

**Table 5:** Correlations between post-COVID-19, visio-spatial abilities, Addenbrooke’s cognitive examination, anxiety, and depression score

Variables	COVID-19	Visio-spatial abilities	Addenbrooke’s Cognitive Examination	Anxiety score
**Visio-spatial abilities**	**0.090** **(*P*= 0.395)**			
**Addenbrooke’s Cognitive Examination**	***R*= − 0.190** **(*P*= 0.069)**	*R*= 0.504^**^ **(*P*< 0.001)**		
**Anxiety score**	*R*= 0.341^**^ **(*P*= 0.001)**	***R*= − 0.170** **(*P*= 0.106)**	*R*= − 0.409^**^ **(*P*< 0.001)**	
**Depression score**	*R*= 0.207^*^ **(*P*= 0.048)**	***R*= − 0.197** **(*P*= 0.060)**	*R*= − 0.283^**^ **(*P*= 0.006)**	*R*= 0.763^**^ **(*P*< 0.001)**

*P value < 0.05 (significant)

**P value < 0.01 (highly significant)

**Table 6:** Correlations between post-COVID-19 and degrees of anxiety and depression

Variables		COVID-19	Anxiety degree
**Spearman’s rho correlation coefficient**	**Anxiety degree**	0.397^**^ **(*P*< 0.001)**	
	**Depression degree**	0.279^**^ **(*P*= 0.007)**	0.783^**^ **(*P*< 0.001)**

**P value < 0.01 (highly significant)

**Table 7:** Correlations between post-COVID-19, age, attention, memory, verbal fluency, and language scores

Variables	COVID-19	Age	Attention	Memory	Verbal fluency
**Age**	***R*= 0.158 (*P*= 0. .133)**				
**Attention**	*R*= − 0.356^**^ **(*P*< 0.021)**	***R*= 0.048** **(*P*= 0.649)**			
**Memory**	*R*= − 0.362^**^ **(*P*< 0.001)**	***R*= − 0.085** **(*P*= 0.421)**	*R*= 0.240^*^ **(*P*= 0. 021)**		
**Verbal fluency**	***R*= − 0.157** **(*P*= 0.135)**	*R*= − 0.255^*^ **(*P*= 0.014)**	***R*= 0.034** **(*P*= 0.745)**	***R*= 0.076** **(*P*= 0.472)**	
**Language**	***R*= − 0.089** **(*P*= 0.396)**	***R*= 0.043** **(*P*=0.681)**	*R*= 0.434^**^ **(*P*< 0.001)**	***R*= 0.136** **(*P*= 0.195)**	***R*= 0.039** **(*P*= 0.709)**

*P value < 0.05 (significant)

**P value < 0.01 (highly significant)

On the other hand, there was no significant statistical correlation between post-infection with COVID-19 and age, language, verbal fluency, visio-spatial abilities, and Addenbrooke’s Cognitive Examination (*p* > 0.05) (Tables 5, 6, and 7).

Multiple linear regressions were used considering memory score as an outcome or predicted variable while post-COVID-19, depression score, and anxiety score as predictors. The result revealed a highly negative strong significant effect of post-COVID-19 on memory scores controlling both anxiety and depression degrees (Beta = − 0.745, *P* < 0.001). Also, there was a strong negative correlation of depression score with memory while controlling for post-COVID-19 and anxiety variables (Beta = − 0.391, *P* = 0.001).

Another multiple linear regression was used considering attention score as an outcome or predicted variable while post-COVID-19, depression score, and anxiety score as predictors. The result revealed a highly strong significant negative effect of post-COVID-19 on attention scores controlling both anxiety and depression degrees (Beta = − 0.745, *P* < 0.001). Also, there was a strong negative correlation of depression scores with attention while controlling for post-COVID-19 and anxiety variables (Beta = − 0.365, *P* < 0.001). On the other hand, anxiety had no significant effect on both memory and attention while controlling for post-COVID-19 and depression score variables (*P* > 0.05).

The total number of participants in this study was 92 healthcare workers; 16 (17.4%) were males and 76 (82.6%) were females. Their ages ranged from 22 to 54 years. The number of post-COVID-19 cases was 32 while the comparative group was 60 healthcare workers with different job categories: 17 physicians, 3 dentists, 36 pharmacists/chemists, 16 nurses, and 20 assistants. The results showed that there were no statistically significant differences between post-COVID-19 cases and comparative groups according to gender and job category (Table 1).

**Fig. 1:**
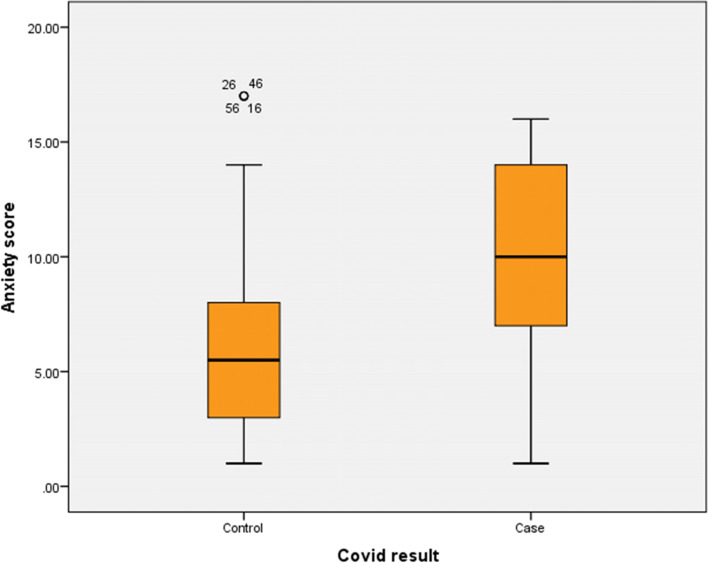
COVID result: anxiety score

**Fig. 2:**
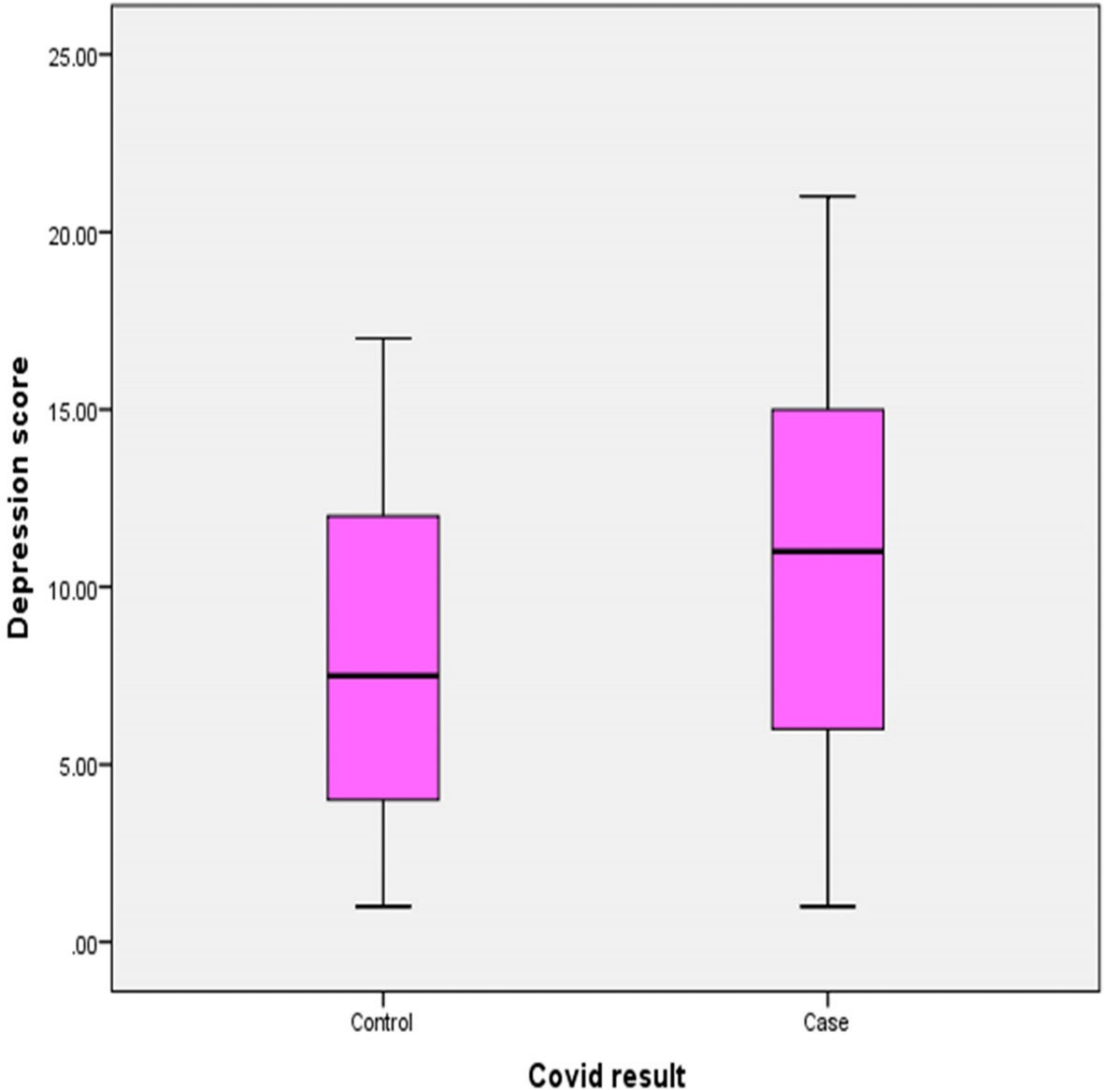
COVID result: depression score

The results showed that there were no statistically significant differences between post-COVID-19 cases and the comparative group according to mean age, verbal fluency, language scores, and visio-spatial abilities. Although the mean score of Addenbrooke’s Cognitive Examination among post-COVID-19 cases was lower than that of the comparative group, the difference was still insignificant (*P* = 0.069). The mean scores of memory and attention were lower among post-COVID-19 cases than those among the comparative group where the differences were highly statistically significant (*p* < 0.01). The mean scores of anxiety and depression among post-COVID-19 cases were significantly higher than the comparative group (*p* = 0.048 and 0.001, respectively) (Table 2 and Figs. 1 and 2).

Concerning the degree of anxiety and depression, the lowest degrees were recorded among the comparative group while the post-COVID-19 cases group recorded the highest ones. The difference between the two groups was highly statistically significant (*p* = 0.002) (Table 3).

The results revealed that there was a highly significant direct correlation between post-infection with COVID-19 and both scores of anxiety and depression (*p* = 0.001 and 0.45, respectively). On other hand, there was no significant statistical correlation between post-infection with COVID-19 and both scores of visio-spatial abilities and Addenbrooke’s Cognitive Examination (*p* > 0.05) (Table 5).

There was a highly significant direct correlation between post-infection with COVID-19 and both scores and degrees of anxiety and depression (*p* < 0.01) (Table 6).

A highly significant inverse correlation was found between post-infection with COVID-19 and both scores of attention and memory (*p* < 0.001). On the other hand, there was no significant statistical correlation between post-infection with COVID-19 and age, language, and verbal fluency (Table 7).

**Fig. 3:**
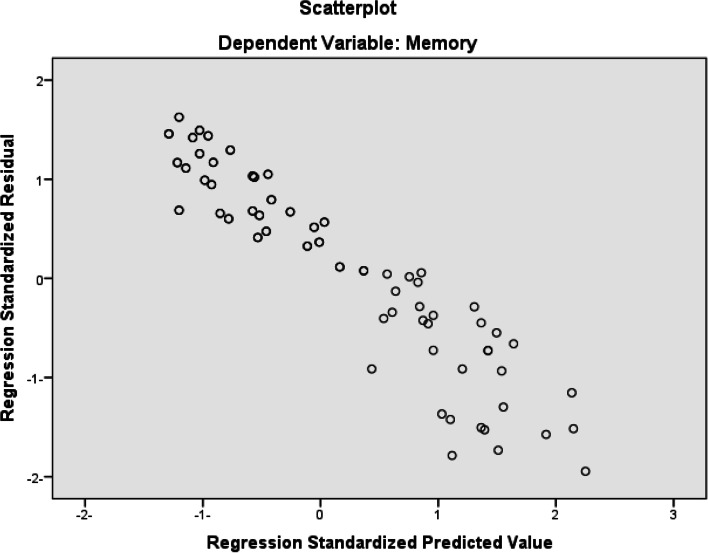
Scatterplot shows dependent variable: memory; regression standardized residual; regression standardized predicted value

A multiple linear regression was used considering memory score as an outcome or predicted variable while post-COVID-19, depression score, and anxiety score as predictors. The result revealed a highly strong negative significant effect of post-COVID-19 on memory scores controlling both anxiety and depression degrees (Beta = − 0.745, *P* < 0.001). Also, there was a strong negative correlation of depression scores with memory while controlling for post-COVID-19 and anxiety variables (Beta = − 0.391, *P* = 0.001) (Table 8 and Fig. 3).

**Table 8:** Multiple linear regression considering memory variable as an outcome while predictors are post COVID-19, depression score, and anxiety score

Predictors (variables)	Standardized coefficients Beta	*t*	***P*** values	95% CI of the coefficient
**Post-COVID-19**	− 0.745	− 9.285	< 0.001	− 14.286, − 9.249
**Depression score**	− 0.391	− 3.321	0.001	− 1.358, − 0.341
**Anxiety score**	− 0.188	− 1.565	0.121	− 0.127, − 1.071

**Fig. 4:**
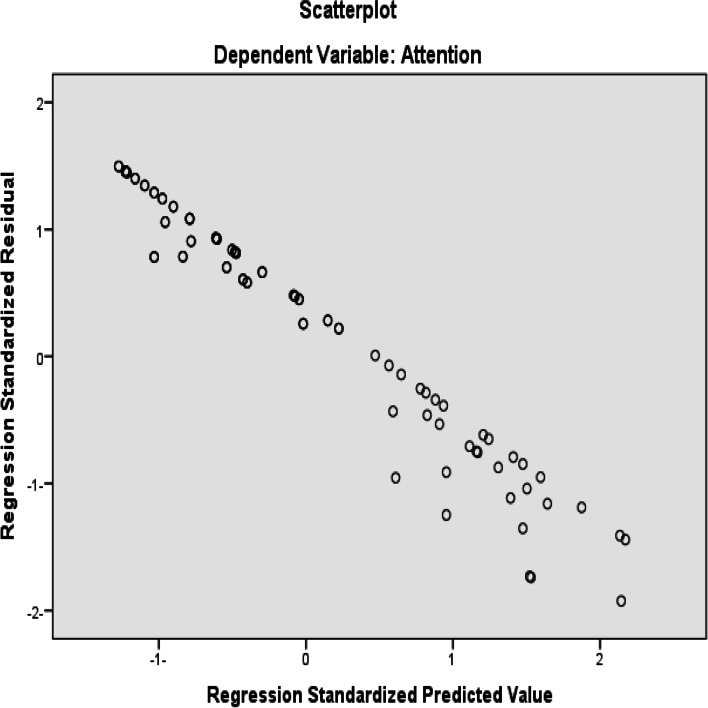
Scatterplot shows dependent variable: attention; regression standardized residual; regression standardized predicted value

Another multiple linear regression was used considering attention score as an outcome or predicted variable while post-COVID-19, depression score, and anxiety score as predictors. The result revealed a highly strong significant effect of post-COVID-19 on attention scores controlling both anxiety and depression degrees (Beta = − 0.745, *P* < 0.001). Also, there was a strong correlation of depression scores with attention while controlling for post-COVID-19 and anxiety variables (Beta = − 0.365, *P* = 0.001). On the other hand, anxiety had no significant effect on memory or attention while controlling for post-COVID-19 and depression score variables (Table 9 and Fig. 4).

**Table 9:** A multiple linear regression considering attention score variable as an outcome while predictors are post-COVID-19, depression score, and anxiety score

Predictors (variables)	Standardized coefficients Beta	***t***	***P*** values	95% CI of the coefficient
**Post-COVID-19**	− 0.745	− 10.268	< 0.001	− 10.798, − 7.296,
**Depression score**	− 0.365	− 3.433	0.001	− 0.964, − 0.257
**Anxiety score**	− 0.146	− 1.345	0.182	− 0.698, − 0.134

## Discussion

Starting in 2019, the world is still facing the COVID-19 pandemic and its complications [1]. Although the infection is originally known to affect the respiratory system [18], literature is rapidly evolving to expose a multifaceted disease mysteriously and collectively named post-COVID with psychiatric [19–21] and neurological complications [6, 22, 23].

One of the commonly reported complaints is that cognitive functions decline after recovering from COVID-19 infection. This impairment was growingly reported in heterogeneous populations [24]. as in the elderly [25], critically ill [26], and hospitalized [27]. Nonetheless, such decline was also reported in everyday talks of recovered healthcare workers. One would talk about how “foggy” he or she became after recovering from COVID-19. Despite this, up to our knowledge, no previous study assessed the cognitive functions in healthcare workers post-COVID-19 infection. This motivated our study to assess cognitive functions using ACE-III in a sample of healthcare workers who recovered from COVID-19 infection in a duration between 2 weeks and 3 months. Our sample strictly included individuals between 22 to 54 years, who were previously healthy, and they have not been admitted either to an intensive care unit or a hospital.

As depressive and anxiety symptomatology is associated with cognitive deficits as well, screening for anxiety and depression symptoms were respectively assessed by the GAD-7 and PHQ-9. The interview was devoid of any information related to post-COVID, to avoid any response bias that could arise, as, in a study by Winter and Braw [28], exposure to information regarding post-COVID neurological symptoms increased self-reported symptoms of cognitive dysfunction.

To eliminate the risk of confounding and in line with the cognitive reserve hypothesis that suggests that higher education, regular participation in mentally stimulating activities, and complexity of occupation increase an individual’s resistance to cognitive decline, our study included matched cases and a comparative group from the same field [29]. Also, the same work conditions would balance the social isolation adverse effects as linked to cognitive decline in the absence of aging covariates [30].

Among the 92 study participants, ACE-III was conducted to test the cognitive domains: attention, memory, verbal fluency, language, and visuospatial abilities. Although the difference in the mean score of ACE-III among post-COVID-19 cases and the comparative group was insignificant, the cases group scored lower. Both anxiety and depressive symptoms scores were significantly prominent in the cases group.

On the one hand, as regards the individual tests, the mean scores of memory and attention were highly significant among cases to reveal a prominent affection. On the other hand, our results indicate that neurocognitive deficits after recovery from COVID-19 are independent of depressive and anxiety symptoms. These findings were reported in analyzed data from 81,337 individuals in which spatial working memory and selective attention deficits were revealed, even after carefully controlling for premorbid IQ, pre-existing medical conditions, socio-demographic factors, and psychiatric symptoms. Those who received mechanical ventilation had the greatest impairment [9]. Although Woo et al. [31] reported that short-term memory, attention, and concentration were particularly affected by COVID-19, their screening results did not correlate with hospitalization, treatment, viremia, or acute inflammation and were independent of depressive symptoms or fatigue.

In a study by Almeria et al., cognitive profiles following COVID-19 infection included lower scores on memory, attention, executive functions, and the global cognitive index, as well as higher scores in anxiety and depression, which were reported in the group with cognitive complaints following COVID-19 infection [32].

Zhou et al. described cognitive dysfunction in the sustained attention domain in COVID-19 patients [33]. While a study on 267 participants, a positive SARS-CoV-2 test was associated with about a 5-times greater likelihood of reporting subjective memory problems at a follow-up after 8 months [34]. In 58% of a cohort aged between 22 and 71 years, a decrease in cognition was observed as reflected by lower scores on Montreal Cognitive Assessment Scale (MoCA) [35]. In a review of objective cognitive tests data from 12 studies, patients with COVID-19 infection between a few days and 6 months have experienced global cognitive impairment. Moreover, some encountered memory and attention problems and impairment in executive functions, particularly verbal fluency [36].

Being in the frontline, being responsible for saving lives, and having long shifts, the ambiance of the crisis would exert an extra burden on the already affected individual. Hence, our result shed light on subjects who recovered from COVID-19; they should undergo long-term monitoring for evaluation of cognitive functions and implement early intervention and neuropsychological rehabilitation programs.

*Limitations* include a cross-sectional study design to assess cognitive function post-COVID-19, although no baseline data was found before the pandemic. The recruited sample was a convenience sample which may limit the generalizability of the results.

*Strengths* include being among the first to tackle the objective assessment of cognitive functions in healthcare workers. Another strength was the matching between the case and the comparative group; from the same field with the elimination of age, gender, and sociodemographic differences.

*Recommendations* are in line with those of Oh, Vannorsdall, and Parker [37]; the need for longer prospective studies that include individuals from different races and ethnicities to determine symptom timelines and whether such impairment is long-lasting or temporary. More research is needed to understand the underlying biological mechanism of the effect of SARS-CoV-2 on the central nervous system

## Conclusions

In our sample of healthcare workers recovering from COVID-19, attention and memory were significantly affected. Depressive and anxiety symptoms were more reported among the cases group, and there is a direct correlation between these symptoms and attention and memory scores. Nonetheless, attention and memory affection were independent of the assessed depressive and anxiety symptomatology. Our findings shed light on the importance of neuropsychological interventions besides the more commonly implemented psychological ones, in the context of care of the carers.

## Data Availability

The data used to support the findings of this study can be found in the manuscript.

## Abbreviations

ACE-III: Addenbrooke’s Cognitive Examination III
COVID-19: Coronavirus infection
GAD-7: Generalized Anxiety Disorder Scale
HCWs: Since healthcare workers
PHQ-9: Patient Health Questionnaire
WHO: World Health Organization
